# Geographic Remoteness, Socioeconomic Status, and Healthcare Access: Emergency Preparedness of South Dakota Secondary Schools

**DOI:** 10.1155/tsm2/4600636

**Published:** 2025-06-07

**Authors:** Trevor C. Roiger, Mary Beth Zwart, Angela E. Brown, Hailey A. Haber, Nicholas R. Anderson

**Affiliations:** School of Health and Human Sciences, South Dakota State University, Brookings, South Dakota, USA

## Abstract

**Context:** Athletic emergency preparedness remains critical to ensure athlete safety.

**Objective:** To assess the influence of geographic remoteness, socioeconomic status, and healthcare access on athletic emergency preparedness of South Dakota secondary schools.

**Design:** A cross-sectional study.

**Setting:** Secondary schools.

**Patients or Other Participants:** A total of 63 athletic directors (age = 44.09 ± 12.39 years, years as AD = 6.96 ± 7.47).

**Main Outcome Measure(s):** A survey assessing athletic training (AT) and emergency medical services (EMSs), emergency action plans (EAPs), and CPR and AED, concussion, and heat illness policies and guidelines. Descriptive statistics were reported. An independent *t* test was performed to determine association between median household income (MHI) and access to ATs. Contingency tables and logistic regression were used to determine if access to an AT, hospital distance, and/or MHI were correlated with question responses.

**Results:** Most respondents (73.03%) reported AT access; access was associated with MHI. Over 20% of schools were farther than 20 miles from nearest hospital. Schools without ATs in low MHI counties were less likely to have written EAPs (*p*=0.03, *n* = 48); schools without ATs were less likely to have EAPs for every venue (*p*=0.027, *n* = 32), CI (−10.7, −0.79). Most respondents (66.7%) reported no CPR certification and AED training requirement for coaches. Schools with ATs reporting farther hospital distance were less likely to have written heat illness guidelines (*p*=0.02, *n* = 36, and CI = −0.17–0.017). Schools in low MHI counties were less likely to possess these same guidelines (*p*=0.04 and *n* = 47) and were less likely to take environmental measurements to inform activity modifications (*p*=0.03 and *n* = 48).

**Conclusions:** Limited AT access, geographic remoteness, and MHI can negatively impact athletic emergency preparedness. Schools may benefit from educational opportunities and emergency preparedness training of coaches and administrators.

## 1. Introduction

Secondary school-sponsored athletics supported nearly 8 million high school participants in 2023-24 [1]. These athletic programs not only enhance participant quality of life but also present an inherent risk of injury [2, 3]. This risk includes catastrophic injuries, those resulting in fatalities, permanent functional disability, or serious, functional disability with full recovery [4]. Between 1982 and 2023, the National Center for Catastrophic Sport Injury Research (NCCSIR) documented 2503 catastrophic events at the high school level. During the 2022-23 school year alone, 76 catastrophic events occurred; this included 15 fatalities [5]. On a regional level, a study of 5 rural Midwest states including South Dakota found a concerning number of sport-related injuries in children under 20 years of age that resulted in hospital emergency room visits, restricted activity longer than 3 months, or persistent functional problems [6]. While state-specific sport injury data are limited, two recent fatalities associated with high school sport participation in South Dakota have illuminated the risk of catastrophic injury in all schools including those located in more geographically isolated states [7, 8]. Catastrophic injuries particularly highlight the importance of risk mitigation efforts including those that focus on well-developed emergency action plans (EAPs). Unfortunately, many secondary schools do not adopt EAPs [9]; when they do, the EAPs vary widely in comprehensiveness and consistently fail to adhere to best practices in emergency planning [10].

Insufficiencies in secondary school EAPs have been linked to various barriers including the absence of athletic training (AT) services, rurality, and lower socioeconomic status [11–14]. Athletic trainers are multiskilled, licensed healthcare providers that complete extensive training in a variety of domains including injury and illness recognition, treatment, and risk reduction [15]. Athletic trainers work in a variety of settings including secondary schools, the majority of which are considered rural [16] Regardless of geographic locale, athletic trainers are instrumental to secondary school emergency preparedness efforts, fostering higher rates of EAP adoption and adherence to EAP best practices including having written, venue-specific plans and timely access to emergency equipment [10]. Unfortunately, access to AT services in secondary schools remains limited, especially in states with comparatively higher rural populations including Montana, South Dakota, North Dakota, Wyoming, and Iowa [16, 17]. Overall, approximately 40% of South Dakota schools report no access to AT services [18]; nearly half of rural South Dakota schools report no access [16]. Schools with AT services, especially in more rural states including South Dakota, most commonly receive these services in a part-time capacity [18]. Delineating between full and part-time AT services is important, especially in rural settings. Rural schools with part-time AT services are less likely to have EAPs compared with suburban schools with full-time services [12].

Though rurality or what it means to be rural can be vague, rural and frontier areas have been categorized in part by population density and geographic remoteness [19]. Individuals in rural and frontier areas live farther away from hospitals, experience longer EMS response times, and suffer poorer health-related outcomes than their urban counterparts [19–21]. A recent study of geographic disparities in distance to trauma care indicated that nearly 50% of secondary schools across Oklahoma, Kansas, Nebraska, Missouri, Iowa, South Dakota, and North Dakota are located greater than 50 miles from a tertiary trauma center [22]. Furthermore, secondary schools located in rural areas more distant from tertiary trauma care centers are less likely to have access to AT services [22]. In South Dakota specifically, 64 out of 66 counties are considered rural or frontier [23]. South Dakota has a single Level I trauma center and 3 Level II trauma centers. These definitive care centers are located in the far Southeast and Western regions of the state [24]. Timely access to definitive care trauma centers critically impacts mortality rates associated with catastrophic events including those affecting pediatric patients [25–27]. Access to healthcare in South Dakota is bolstered through 40 Critical Access Hospitals [28]; these hospitals are located at least 35 miles apart, maintain a maximum of 25 in-patient beds, and provide 24-h emergency care services. These hospitals are designed to provide essential healthcare services to rural communities; however, the nature of their 24-h emergency care services remains highly variable [28]. Distance to hospitals in South Dakota and similarly rural states is complicated by access to ambulance services. Research indicates that over 4 million individuals across the U.S. live in ambulance deserts; most of these individuals live in more rural counties [29]. Over 85% of South Dakota counties maintain populations residing in areas located at least 25 min from an ambulance service [30]. Multiple western and midwestern states including Wyoming, Montana, and North Dakota report fewer than 3 ambulance services covering every 1000 square miles of land area [29]; currently, there are fewer than 2 ambulance services per 1000 square miles across South Dakota [30]. Correspondingly, South Dakota's EMS response times range from 13 to 21 min and average over 16 min [31].

Socioeconomic challenges also influence emergency preparedness, especially in rural areas. Rural communities often experience lower incomes and higher rates of poverty [12, 20]. South Dakota ranks in the lowest third nationally in median household income (MHI) [32]. Income impacts health-related outcomes and quality of life through access to healthcare services [12, 32, 33]. Secondary schools in counties with lower MHI have reported lower levels of AT access [12]. Similarly, a recent study of emergency action planning in U.S. high schools found that high poverty schools (identified by percentage of students qualifying for free and reduced-price lunch [FRPL]) were less likely to have access to AT services and were less likely to possess EAPs [34]. Income also affects ambulance availability; South Dakota's ambulance services remain largely staffed by volunteers who maintain full-time employment outside of their EMS role [30].

Knowledge of secondary school athletic emergency preparedness specific to the more sparsely populated, geographically isolated rural areas of the Midwest and Great Plains region of the United States is currently lacking. In South Dakota specifically, secondary school athletic emergency preparedness has not been investigated and reported. Geographic isolation and socioeconomic challenges can adversely affect health-related outcomes and may complicate emergency preparedness for rural secondary schools including those in South Dakota. Furthermore, with many secondary schools in rural areas including those found across South Dakota lacking access to AT services [18, 22], the responsibility of emergency preparedness falls on school administrators and coaches who may not possess the knowledge or training to respond appropriately to athletic emergencies [11]. In order to guide emergency preparedness in rural secondary schools, factors that may influence adoption of best-practice EAPs and emergency preparedness policies and procedures must be investigated. Therefore, the purpose of this study was to assess the influence of AT services, geographic remoteness, and socioeconomic status on athletic emergency preparedness of South Dakota secondary schools.

## 2. Methods

### 2.1. Participants

All South Dakota secondary school athletic directors (*n* = 177) were invited to participate in this cross-sectional study by completing an online survey related to athletic emergency preparedness. Athletic director email addresses were obtained from https://gobound.com, a publicly accessible website providing demographic data for all school districts across South Dakota. Prior to data collection, the South Dakota State University Institutional Review Board granted the study exempt status. Athletic directors provided their consent by clicking on the hyperlinked survey in the invitation email. This study, including participant eligibility and selection, followed the recommendations of Strengthening the Reporting of Observational Studies in Epidemiology (STROBE) (STROBE-checklist-v4-combined-PlosMedicine).

### 2.2. Instrumentation

A 54-item survey was employed to assess athletic directors' perceptions of secondary school emergency preparedness. Our survey was based on a previous survey of Arizona secondary school athletic directors' perceptions of emergency preparedness; this survey was developed, assessed, revised, and ultimately approved by the Arizona Interscholastic Association's Sports Medicine Advisory Committee [35]. This same survey, with appropriate language modifications made to reflect state-specific language, was utilized in a study of secondary school emergency preparedness in Iowa [13]. Similarly, questions on our survey were modified to reflect current policies or requirements specific to South Dakota secondary schools and also reflected best practices in emergency planning from the National Athletic Trainers' Association [36]. The survey included binary (*yes* or *no*), multiple choice, and multipart, closed-ended items related to AT services, emergency preparedness, CPR and AED, concussion, and heat illness policies and guidelines, access to emergency equipment, and EMS services, specifically distance and drive time to nearest hospital. The survey was reviewed for clarity by 2 experts, a former secondary school athletic trainer and member of South Dakota's Sports Medicine Advisory Committee, and a South Dakota secondary school athletic director with more than 5 years of experience. Based on feedback from these individuals, no adjustments were made to the survey. Socioeconomic data, specifically host county MHI, were identified through the South Dakota Department of Health. Athletic directors were asked demographic questions related to their experience in the athletic director role as well as their school's athletic classification. The survey was distributed electronically via QuestionPro.

### 2.3. Procedures

We emailed all South Dakota secondary school athletic directors requesting their voluntary participation in this survey. The email described the purpose of the study and included a link to the survey. Athletic directors were informed that the survey would take approximately 15–20 min to complete. Data collection occurred between August-September of 2023. Athletic directors were initially emailed in August and nonrespondents received up to 3 follow-up emails at 2-week intervals.

### 2.4. Data Analysis

Statistical analysis was done using R 4.3.1. All responses were included in the data analysis whether a survey was initiated or completed in whole. Descriptive statistics for survey items were reported by percentage and frequency. Athletic director demographics were characterized by the mean ± SD or percentage and frequency. Survey logic was used to allow for follow-up questions if a respondent answered a question in a particular manner; this led to variations in the number of responses per question. An independent *t* test was calculated to investigate differences in county MHI between schools with AT services and those without. Questions with a binary response were analyzed using contingency tables and logistic regression to determine if access to an AT, distance to hospital, and/or MHI were correlated with the response to the question. Hierarchical clustering was used to determine high and low MHI. Contingency tables were created for the response to the question and each of the covariates. A fisher exact test was then used to determine if there were statistically significant (*p* value < 0.05) differences in response based on the covariates in question. For questions in which statistical significance existed using the fisher exact test, a logistic regression model was fit to determine the direction of the relationships between the covariates and the response. Any observation that had missing data with respect to the outcome and predictor variables for a given model was automatically removed. The direction, *p* value, and 95% confidence interval are reported for each of the estimates of the logistic regression model which were significant as well as *p* values from the fisher exact tests for the contingency tables.

## 3. Results

### 3.1. Response Rate, Athletic Director, and School Characteristics

Across all South Dakota secondary school athletic directors (*n* = 177), 112 started (63.3%) and 63 completed (35.6%) the survey. The athletic directors were 44.09 ± 12.39 years of age and possessed 6.96 ± 7.47 years of overall experience as an athletics administrator (Table 1).

### 3.2. AT and EMS Access

Most respondents (73.03%, *n* = 65/89) reported their school had access to AT services; of these, over 75% (*n* = 48/63) indicated the services were part-time (anything less than 30 h per week, 5 days per week and 10 months per year). More than 45% (*n* = 21/46) indicated their part-time services included game coverage only. Approximately half of the schools (52%, *n* = 13/25) have access to other medical providers for practice and/or game coverage, mostly local EMS (61.54%, *n* = 8/13) and largely for football game coverage only (92.31%, *n* = 12/13). Over 70% (*n* = 48/68) of the respondents indicated the average distance in miles between their school's practice and game venues and the nearest hospital was 15 miles or less. Two-thirds of the respondents reporting their school district and county information (*n* = 30/45) were employed in schools more than 50 miles from the nearest tertiary, definitive care trauma center. Nearly 30% of these schools (*n* = 13/45) were located in an EMS district with an average dispatch to arrival on scene time of 19.2 min (range = 17.17–21.65); a majority of these schools (*n* = 7/13) reported no AT services. Most school districts (60.0%, *n* = 27/45) were in low MHI counties (Table 2).

### 3.3. Emergency Planning

Over 76% of the respondents (*n* = 58/76) reported having a written EAP for their school. Schools without AT services that were in low MHI counties were less likely to have a written EAP (*p*=0. 03 and *n* = 48 from the contingency table). While nearly 91% (*n* = 49/54) of the respondents indicated having a written plan for every practice and game venue, schools without access to AT services were less likely (*p*=0.027) from logistic model with *n* = 32 and CI (−10.7, −0.79). Fewer than half of the respondents (41.51%, *n* = 22/53) reported annually distributing and reviewing their school's EAP. Nearly three-fourths of schools (74.1%, *n* = 40/54) do not practice the EAP with critical personnel on an annual basis. School administrators (34.15%, *n* = 14/41) and coaches (29.27%, *n* = 12/41) were most involved in practicing the EAP. While local EMS (12.2%, *n* = 5/41) was less involved with annual EAP practice, respondents overwhelmingly (92.16%, *n* = 47/51) reported that EMS was familiar with their schools' practice and game venues to ensure efficient emergency response.

### 3.4. CPR and AED Access and Preparedness

Each respondent (100%, *n* = 63/63) reported access to an automated external defibrillator (AED) at the school; approximately 62% (*n* = 40/65) reported 3 or more. Most respondents (85.94%, *n* = 55/64) indicated that 100% of the school's practice and game venues had access to an AED within 3–5 min. One-third (*n* = 22/66) of he respondents reported their school required coaches to be CPR certified and trained in the use of AEDs; half (*n* = 28/56) indicated this applied to head coaches only. Over 12% (*n* = 8/66) indicated their school had no access to AT services and no requirement for coach CPR certification and AED training. Of these, 3 reported greater than 20 miles to the nearest hospital. There was no significant difference in any of the preceding findings based on access to AT services, distance from hospital, or MHI.

### 3.5. Concussion and Heat Illness Policies and Procedures

There were no significant differences in concussion policies and guidelines based on access to AT services, MHI, or distance from hospital. Most respondents (95.45%, *n* = 63/66) reported familiarity with the South Dakota High School Activities Association (SDHSAA) concussion guidelines and information, with 75% (*n* = 48/64) indicating their school provides concussion education training to relevant stakeholders on an annual basis. Nearly 80% (*n* = 51/65) of the respondents reported having written concussion guidelines for the school; over 68% (*n* = 42/61) reported having a school board approved concussion policy. Most respondents (84.38%, *n* = 54/64) reported taking environmental measurements before practices and games to inform activity modifications. Heat index (*n* = 49, 28.99%), air temperature (*n* = 46, 27.22%), lightning (*n* = 45, 26.63%), and WGBT (*n* = 28, 16.57%) were the reported environmental measurements taken before practices and games.

Most respondents (93.65%, *n* = 59/63) reported familiarity with the SDHSAA heat illness policy, with slightly over half (51.61%, *n* = 32/62) possessing written heat illness guidelines for their school. Schools with access to AT services and greater hospital distance were less likely to possess written heat illness guidelines based on logistical regression (*p*=0.02, *n* = 36, and CI = −0.17–0.017). Based on the contingency table, schools located in lower MHI counties were less likely to possess these same guidelines (*p*=0.04 and *n* = 47). Nearly 85% of schools (*n* = 54/64) take environmental measurements before practices and games to inform activity modifications; schools located in lower MHI counties were less likely to do so (*p*=0.03 and *n* = 48 from contingency table). Over two-thirds of the respondents reported their school (*n* = 42/62) did not have a written treatment plan for heat illness and more than half (57.89%, *n* = 11/19) had no access to cold water immersion (CWI) within 3–5 min of practice and game venues. Of the 11 schools reporting no access to CWI, 5 also reported no access AT services; one of these 5 reported greater than 20 miles to nearest hospital.

## 4. Discussion

### 4.1. AT and EMS Access

Our results indicate that almost three-fourths of South Dakota secondary schools receive AT services. Though higher than previously reported [18], our results could stem from the fact that the strong majority of responses came from ADs employed at secondary schools in the more densely populated, less rural Eastern third of South Dakota. Interestingly, over 75% of AT services in our study were identified as part-time; this is considerably higher than previously reported [18]. While our results could relate to changing dynamics in accessibility to AT services, the prevalence of part-time AT services in our study remains a concern. Though the definition used for part-time services appropriately follows existing literature [18], how part-time services are defined leaves a wide range of variability in potential time spent at a school. This is supported by nearly half of our study respondents indicating that their school's part-time AT services entailed game coverage only. We contend that reliance on part-time AT services may be insufficient for secondary school emergency preparedness as schools with part-time services have been shown to adopt fewer EAP best practices than schools with full-time AT services [10]. Furthermore, the level of part-time AT services as measured by hours per week has been shown to vary widely [12]. Part-time services that are extremely limited may still leave a large portion of young athletes without timely AT access at practices and/or games. This is especially concerning given that secondary schools with no access to AT services have reported a nearly 42% decreased incidence of EMS activations as compared with schools with full-time AT services [37]. This could mean that individuals charged with first response to athlete injuries and illnesses at schools without AT services, typically coaches or administrators, may not recognize the potential urgency of a medical situation and the need for advanced care, putting these athletes at risk for adverse outcomes.

AT services were associated with MHI. This supports prior work by Post et al. [12] and Hedberg et al. [34] who utilized county MHI and/or percentage of students enrolled in FRPL programs as socioeconomic indicators, comparing schools with and without AT services. For our study, we utilized county MHI and did not include FRPL as a SES indicator. Schools with access to AT services were located in counties with MHI nearly $10,000 greater than schools with no AT access. The majority of respondents reporting their school district and county information (60%) were located in low MHI counties. Secondary schools who sponsor athletic programs have a responsibility to ensure the health and safety of student athletes. As such, schools without AT access in higher poverty areas should consider collaborating with proximate districts to provide AT services. Alternatively, partnering with local healthcare organizations may also provide opportunities to receive AT services in an outreach capacity.

While just over half of schools reported access to other healthcare professionals for medical coverage, this access was predominantly for football games only. In addition, nearly 1 out of 5 schools in this study were farther than 25 miles from the nearest hospital, with almost 44% residing more than 50 miles from a tertiary, definitive care trauma center. Our finding of distance to hospitals, in particular Level I or II trauma centers, is similar to findings from across District 5 of the National Athletic Trainers' Association [22]. This is important to note considering the strong majority of responses came from ADs at schools with some level of access to AT services and located in the more densely populated eastern third of South Dakota. This finding is also concerning given that schools farther from tertiary trauma centers were less likely to have access to AT services [22]. Importantly, limited access to AT and timely access to hospitals and ambulance services characteristic to rural areas including South Dakota [22, 30, 31] places a higher responsibility on school administrators and coaches who are often underprepared [11] to provide emergency care in cases of catastrophic injury or illness. Collectively, our findings related to distance to hospital are of grave concern as geographic isolation and rurality remain critical moderators for catastrophic injury outcomes [25–27].

### 4.2. Emergency Planning

Best practices in secondary school emergency preparedness include having written EAPs that are annually distributed, reviewed, and rehearsed with critical personnel [36]. Though not associated with distance to hospital, schools that were located in low MHI counties and who did not have access to AT services were less likely to possess written EAPs. This finding was consistent with previous research on secondary school emergency preparedness and access to AT services [10, 12–14]. Lower levels of venue-specific planning in low MHI schools could also stem from athletic directors assuming multiple roles, thus leaving less time for EAP development. While the strong majority of athletic directors reported having EAPs that were venue specific, schools without access to AT services were less likely to have these plans. The limitations on venue-specific EAPs in schools lacking access to AT services supports prior work indicating lower levels of EAP adoption in secondary schools without AT services [13, 34]. Requiring school districts to annually submit their venue specific EAPs to SDHSAA may be a step to improving emergency preparedness in school districts without AT services. Another solution would be for school districts to partner with geographically located healthcare centers to develop hiring practices that would improve full-time AT services.

Although not associated with access to AT services, distance from hospital, or MHI, less than half of the schools distribute and review their EAPs on an annual basis and even fewer practice the EAP with critical personnel. The failure to both distribute and review the EAP on an annual basis is similar to previous work in Iowa secondary schools [13]. Annual review of EAPs is critical to ensure accurate information and that the plan continues to meet the needs of the venue. Similarly, distribution of an EAP to all relevant stakeholders ensures thorough understanding of roles, equipment availability, communication, and implementation procedures [37]. Annual rehearsal of EAPs, including with local EMS, remains a prominent gap in EAP best practices for secondary schools [10, 13]. Ultimately, EAPs with lower levels of adherence to best practices place participants and school districts at significant risk for adverse outcomes.

### 4.3. CPR and AED Access and Preparedness

Although none of our findings were associated with access to AT services, distance from hospital, or MHI, several key findings were revealed. All of our respondents indicated that they had 1 or more AEDs at their school, with a majority (85.94%) reporting access to an AED within 3–5 min of each venue. In a comparative study, Williams et al. [13] found that 100% of the schools in Iowa had access to an AED with 64.9% available within 3–5 min. Promptness in defibrillation is vital to survivability in young athletes, with survival rates decreasing every minute of delayed AED application [38]. Respondents' reports of AED access suggest a high degree of preparation for potential cardiac emergencies in South Dakota secondary schools.

Having trained personnel able to provide immediate emergency care is just as crucial to athlete survivability. While the SDHSAA requires coaches to complete NFHS “Collapsed Student” and “First Aid, Health, and Safety” online training every 2 years, these training modules are not designed or intended to take the place of hands-on CPR and AED training. The SDHSAA does not require coaches to complete any kind of hands-on first aid, CPR, or AED training. Only 33% of the respondents in this study indicated that their school requires coaches to be CPR certified and trained in the use of AEDs in addition to meeting the SDHSAA requirements; half of these respondents indicated this applied to head coaches only. Our findings were considerably lower than comparable studies from Iowa and Arizona citing the percentage of secondary school coaches (78.1% and 86%, respectively) who were both CPR certified and trained in the use of AEDs [13, 35]. Student athletes in secondary schools who do not receive AT services depend on coaches and administrators for emergency care. The lack of requirements for CPR certification and AED training for South Dakota coaches places participants at significant risk for catastrophic outcomes in the event of cardiac compromise. Alarmingly, 3 schools in our study reported that they had no access to AT services, did not require their coaches to be CPR certified and AED trained, and were farther than 20 miles from the nearest hospital. Extended EMS response times across South Dakota also highlight the importance of CPR certification and AED training for first responders, namely secondary school coaches and administrators. Currently, South Dakota-codified law [39] provides civil immunity for emergency use of AEDs for any person who in good faith uses or attempts to use an AED for emergency care. Previous research has indicated the value of state-level emergency preparedness policy on adoption of EAPs with CPR/AED requirements [40]. Mandates from the SDHSAA, as well as education of secondary school administrators about statutory protections provided by South Dakota law, may lead to a higher percentage of schools requiring coaches to complete hands-on training in CPR and the use of AEDs.

## 5. Concussion

South Dakota concussion law requires the SDHSAA to develop guidelines to educate and inform schools, coaches, athletes, and parents/guardians of the nature and risks of concussions. This includes a concussion information sheet, signed annually by both athlete and parent/guardian, which is included as a part of every athlete's preparticipation requirements. Concussion information sheets include a description of the nature and risks of concussion, signs, symptoms, and behaviors associated with concussions, need for recognition and appropriate referral, and the need to follow medical direction for treatment and return to play. South Dakota concussion law also requires coaches to complete annual concussion training and also describes requirements for removal from play and clearance from a licensed healthcare provider before returning to athletic activities. Concerningly, 3 respondents in our study were not familiar with the SDHSAA concussion guidelines. Over 20% of the respondents indicated their school did not have written concussion guidelines, and just over 30% did not have a school board-approved concussion policy. These findings are lower than those reported in comparable studies [13, 35] and suggest that gaps may exist in schools' compliance with South Dakota concussion law and best practices in concussion recognition and management. Without guidelines or policy in place, schools who lack AT services, reside in lower income regions, or are more distant from healthcare may struggle to ensure that concussed athletes are able to receive appropriate treatment and clearance from a licensed healthcare provider with experience in concussions.

### 5.1. Heat Illness

According to the SDHSAA and Administrative Rules of South Dakota, all Fall sport coaches are required to annually complete the NFHS Heat Illness Prevention program. The SDHSAA also maintains heat acclimatization regulations for football, soccer, and tennis. Concerningly, 4 respondents indicated that they were not familiar with SDHSAA policy regarding heat illness. Exertional heat illnesses (EHIs) can arise in more than just warm, hot, and/or humid conditions. Requiring all coaches regardless of sport and season to complete Heat Illness Prevention training would be advantageous considering the catastrophic potential of EHI.

Approximately half of the schools in our study possess written heat illness guidelines, though schools with AT services and more distant from hospitals, as well as those in lower MHI counties, were less likely. These findings could be explained by limited part-time AT services or athletic directors maintaining multiple roles within their job leaving little time for emergency preparedness. Over 70% of the respondents reported no school board-approved heat illness policy; this is slightly higher than found in Iowa secondary schools [13]. Together, the lack of guidelines and local policy in South Dakota secondary schools, especially those that are more distant from hospitals, may adversely affect prevention and management efforts for EHI, leading to poorer outcomes. Given these results, there is a need to educate school districts on best practice recommendations for recognizing and managing EHI. Education must include the use of CWI tubs to cool first and transport second for cases of exertional heat stroke (EHS). In addition, school districts should be made aware of other cost-effective CWI measures that could be utilized such as a tarp for the TaCO method or body bags for an impervious shroud [41].

Following SDHSAA recommendations, most athletic directors reported their school takes environmental measurements before games and practices to inform activity modifications. Schools in lower MHI counties were less likely to take environmental measurements which could again speak to personnel maintaining multiple job responsibilities, thus lessening the time available for emergency preparedness.

The majority of the respondents in our study reported not having a written treatment plan for heat illness and no access to CWI within 3–5 min of all practice and game venues. Concerningly, 5 respondents reported their school did not have AT services or access to CWI; each of these schools was located in an EMS district with average response times exceeding 17 min. The catastrophic nature of EHI, specifically EHS, has been well documented in secondary school athletics [42]. Best practices in recognition and management of EHS include identification of core temperature via rectal thermometry and urgent access to full-body CWI. Nearly 50% of the respondents in our study reported their school did not take core temperatures in cases of suspected EHI; of those that did, only 1 assessed core temperature via rectal method. These deficiencies combined with a lack of access to CWI place both patients and schools at significant risk.

### 5.2. Limitations

Our survey response rate exceeded 35% based solely on email contact with ADs. The use of follow-up telephone calls to emailed nonrespondents may have improved this rate. While our study is broadly applicable to secondary schools in highly rural states, our results are specific to South Dakota. As such, generalizations of our findings to schools in other states must be carefully considered. Furthermore, our results were based information provided by athletic directors only. Though not validated, the survey utilized for this study was based on 2 previous similar studies that progressed through a rigorous review process and were deemed acceptable for the study outcomes [13, 35]. Although 2 experts reviewed our survey, robustness of the survey may have been improved with additional reviewers. Although the definition of part-time AT services was modeled after previous research [18], this definition leaves a wide range of variability in potential time spent at a given school. Schools with lower levels of part-time AT services may possess emergency preparedness policies and procedures that are similar to schools with no AT services. While MHI as a socioeconomic indicator for income was based on previous research [12], property tax valuation might be more appropriate to an agricultural focused state like South Dakota. Property tax valuations are a component of the public-school funding formula and contribute substantially to the South Dakota economy. Finally, South Dakota is divided into 7 different EMS districts that differ in geographical size and population density. Thus, average EMS response time for a district may not accurately reflect the actual response time for all schools in that district.

### 5.3. Implications and Future Research

The impact of AT services, geographic isolation, and socioeconomic status on emergency preparedness of secondary schools is evident [12, 13, 22, 34, 35]. Rural areas in particular are critically influenced by these elements and are at higher risk for poor outcomes after catastrophic sport-related events [22, 34]. Considering these challenges, administrators and secondary school coaches in South Dakota need to become well versed in best practices for EAPs. Schools would benefit from identifying an emergency planning coordinator responsible for all EAP development and implementation procedures. Healthcare systems, county extension services, or the SDHSAA should consider a more active approach to educating secondary schools on best practices in emergency preparedness. Schools should commit to annual EAP training days that involve all coaches, school administrators, and local EMS. Future research should include novel approaches to EAP education as well as the impact of volunteer EMS services on secondary school emergency preparedness. Lower population, higher poverty states including South Dakota often rely on volunteer EMS services, especially in more rural areas. Unfortunately, smaller communities in less population dense areas are increasingly facing EMS workforce shortages due to a variety of reasons including aging and attrition of current providers, new, younger providers who are less available and/or less likely to volunteer, or barriers to training [30]. These challenges may mean that secondary schools in these communities are less likely to develop and implement best practices in emergency preparedness, especially since there is a high likelihood that these schools also do not have access to AT services.

Given the limitations on access to AT services and the geographic isolation of many South Dakota communities, secondary schools should strongly consider developing policies that require all coaches to secure and maintain hands-on CPR and AED training. A SDHSAA mandate requiring CPR certification and AED training for secondary school coaches would enhance participant safety. Future research should include barriers to coach CPR certification and AED training in South Dakota.

Some respondents reported little experience in an athletic director role. Considering that not all athletic directors were familiar with SDHSAA concussion guidelines, policies, and regulations, schools may need to consider stronger on-boarding processes for new athletic directors. The SDHSAA should consider expanding the requirement for heat illness prevention training to all coaches regardless of season. Schools should adopt policies and procedures that guide their approach to EHI. This should include timely access to CWI for all practice and game venues and a commitment to assessing core temperature in cases of suspected EHI. Future research efforts should include barriers to best practices in EHI recognition and management including the use of rectal thermometry and CWI accessibility.

## Figures and Tables

**Table 1:** Athletic director (AD) demographics.

Variable	Mean ± SD (y)	Range (y)
Age	44.09 ± 12.39	23–72
Years of experience as an AD	6.96 ± 7.47	0.25–32
Years in current role	6.21 ± 7.21	0.2–28

*Note:* Over 35% of respondents (*n* = 20/57) reported holding multiple roles at their school.

**Table 2:** School profiles of athletic trainer (AT) access, geographic remoteness, and median household income (MHI).

Variable	No. (%)
AT access	
Yes	65 (73.0)
No	24 (27.0)
Nature of AT access	
Full-time	15 (23.8)
Part-time	48 (76.2)
Geographic remoteness: distance to nearest hospital (mi)	
< 5	24 (35.3)
5–10	9 (13.2)
11–15	15 (22.1)
16–20	3 (4.4)
21–25	12 (17.7)
> 25	
County MHI	
High	18 (40.0)
Low	27 (60.0)

*Note:* Schools with access to AT services had higher MHI compared with schools without these services ($65024.0 ± $9649.0 versus $55549.7 ± $12015.9; *p* < 0.026).

## Data Availability

Research data are not shared.

## Supporting Information

Supporting Information 1: Supporting File 1: STROBE Checklists—Items that should be included in reports of observational studies. This checklist summarizes the study objectives, methodology including study design, participants, variables and measurement, and statistical methods, and results. A summary of the results to include an interpretation of findings and study limitations is included.
