# Supplementary material for: Geographic Remoteness, Socioeconomic Status, and Healthcare Access: Emergency Preparedness of South Dakota Secondary Schools
Source: Transl Sports Med. 2025 Jun 7;2025:4600636. doi: 10.1155/tsm2/4600636 (PMC12170087; doi:10.1155/tsm2/4600636)
Supplement: Supporting Information — Additional supporting information can be found online in the Supporting Information section. [file 4600636.f1.docx]

STROBE Statement—checklist of items that should be included in reports of observational studies

|  | | Item No. | | Recommendation | Page  No. | | Relevant text from manuscript |
| --- | --- | --- | --- | --- | --- | --- | --- |
| **Title and abstract** | | 1 | | (*a*) Indicate the study’s design with a commonly used term in the title or the abstract | 2 | | Line 28: Design: Cross-sectional study. |
|  |  |  |  | (*b*) Provide in the abstract an informative and balanced summary of what was done and what was found | 2 | | Lines 25-45: Setting: Secondary schools.  Patients or Other Participants: 63 athletic directors (age = 44.09 + 12.39 years, years as AD = 6.96 + 7.47).  Main Outcome Measure(s): …athletic training (AT) and emergency medical services (EMS), emergency action plans (EAPs), and CPR and AED, concussion, and heat illness policies and guidelines…Descriptive statistics…Independent t test was performed to determine association between median household income (MHI) and AT access…Contingency tables and logistic regression…if access to an AT, hospital distance, and/or MHI were correlated with question responses…Results: … |
| Introduction | | | | | | |  |
| Background/rationale | | 2 | | Explain the scientific background and rationale for the investigation being reported | 3-4 | | Line 49-56:… athletics supported nearly 8 million high school participants in 2023-24…. these athletic programs … present an inherent risk of injury… risk mitigation efforts including those that focus on well-developed emergency action plans (EAPs). … most secondary schools do not adopt EAPs…; when they do… vary widely in comprehensiveness and consistently fail to adhere to best practices… Insufficiencies …linked to various barriers including the absence of athletic training (AT) services, rurality, and lower socioeconomic status. |
| Objectives | | 3 | | State specific objectives, including any prespecified hypotheses | 6 | | Line 123: …purpose of this study was to assess the influence of athletic training services, geographic remoteness, and socioeconomic status on athletic emergency preparedness of South Dakota secondary schools. |
| Methods | | | | | | |  |
| Study design | | 4 | | Present key elements of study design early in the paper | 6 | | Line 128: All South Dakota secondary school athletic directors (n=177) were invited to participate in this cross-sectional study by completing an online survey… |
| Setting | | 5 | | Describe the setting, locations, and relevant dates, including periods of recruitment, exposure, follow-up, and data collection | 7 | | Line 153: Procedures:  We emailed all South Dakota secondary school athletic directors…email described the purpose of the study and included a link to the survey… Data collection occurred between August-September of 2023…initially emailed…and received up to 3 follow up emails at two-week intervals. |
| Participants | | 6 | | (*a*) *Cohort study*—Give the eligibility criteria, and the sources and methods of selection of participants. Describe methods of follow-up  *Case-control study*—Give the eligibility criteria, and the sources and methods of case ascertainment and control selection. Give the rationale for the choice of cases and controls  *Cross-sectional study*—Give the eligibility criteria, and the sources and methods of selection of participants | 6 | | Line 128: …all South Dakota secondary school athletic directors… email addresses were obtained from gobound.com… |
|  |  |  |  | (*b*) *Cohort study*—For matched studies, give matching criteria and number of exposed and unexposed  *Case-control study*—For matched studies, give matching criteria and the number of controls per case |  | |  |
| Variables | | 7 | | Clearly define all outcomes, exposures, predictors, potential confounders, and effect modifiers. Give diagnostic criteria, if applicable | 6-7 | | Line 143: … binary, multiple choice, and multipart items related to athletic training services, emergency preparedness, CPR and AED, concussion, and heat illness policies and guidelines, access to emergency equipment, and EMS services, specifically distance and drive time to nearest hospital… if access to an AT, distance to hospital, and/or median household income (MHI) were correlated with the response to the question. |
| Data sources/ measurement | | 8* | | For each variable of interest, give sources of data and details of methods of assessment (measurement). Describe comparability of assessment methods if there is more than one group | 6-7 | | Line 135: A 54-item survey was employed to assess athletic directors’ perceptions of secondary school emergency preparedness… survey was based on a previous survey of Arizona secondary school athletic directors’ perceptions of emergency preparedness…this survey was developed and approved by the Arizona Interscholastic Association’s Sports Medicine Advisory Committee...Questions on our survey were modified to reflect current policies or requirements specific to South Dakota secondary schools…included binary (yes or no), multiple choice, and multipart, closed-ended items related to athletic training services, emergency preparedness, CPR and AED, concussion, and heat illness policies and guidelines, access to emergency equipment, and EMS services…reviewed for clarity by a secondary school athletic director and a former member of the SD Sports Medicine Advisory Committee…MHI, were identified through the South Dakota Department of Health. |
| Bias | | 9 | | Describe any efforts to address potential sources of bias | 7 | | Line 96 and 104: …survey was based on a previous survey of Arizona secondary school athletic directors’ perceptions of emergency preparedness…developed and approved by the Arizona Interscholastic Association’s Sports Medicine Advisory Committee…(our) survey was reviewed for clarity by a former secondary school athletic trainer and member of South Dakota’s Sports Medicine Advisory Committee South Dakota secondary school athletic director with more than 5 years of experience…” |
| Study size | | 10 | | Explain how the study size was arrived at | 6 | | Line 128: All South Dakota secondary school athletic directors (n=177) were invited… |
| Quantitative variables | 11 | | Explain how quantitative variables were handled in the analyses. If applicable, describe which groupings were chosen and why | | 6-7 | Line 160-177: Descriptive statistics for survey items were reported by percentage and frequency…  Survey logic was used to allow for follow-up questions if a respondent answered…in a particular manner…this led to variations…responses per question. Questions with a binary response were analyzed using contingency tables and logistic regression to determine if access to an AT, distance to hospital, and/or median household income (MHI) were correlated with the response to the question. | |
| Statistical methods | 12 | | (*a*) Describe all statistical methods, including those used to control for confounding | | 6-7 | Line 160-177: Statistical analysis was done using R 4.3.1. Descriptive statistics for survey items were reported…demographics were characterized by mean + SD or percentage and frequency… Questions with a binary response were analyzed using contingency tables and logistic regression to determine if access to an AT, distance to hospital, and/or median household income (MHI) were correlated with the response…hierarchical clustering … to determine high and low MHI. Contingency tables were created for the response to the question and each of the covariates…A fisher exact test…to determine…statistically significant (p-value < 0.05) differences in response based on the covariates…for questions in which statistical significance existed… logistic regression model was fit to determine the direction of the relationships between the covariates and the response... The direction, p-value, and 95% confidence interval are reported… as well as p-values from the fisher exact tests for the contingency tables... independent t test was calculated to investigate differences in county median household income (MHI) between schools with AT services and those without…Chi-Square analysis was performed …association between high or low MHI and access to AT… | |
|  |  |  | (*b*) Describe any methods used to examine subgroups and interactions | |  |  | |
|  |  |  | (*c*) Explain how missing data were addressed | |  |  | |
|  |  |  | (*d*) *Cohort study*—If applicable, explain how loss to follow-up was addressed  *Case-control study*—If applicable, explain how matching of cases and controls was addressed  *Cross-sectional study*—If applicable, describe analytical methods taking account of sampling strategy | |  |  | |
|  |  |  | (*e*) Describe any sensitivity analyses | |  |  | |
| Results | | | | | | | |
| Participants | 13* | | (a) Report numbers of individuals at each stage of study—eg numbers potentially eligible, examined for eligibility, confirmed eligible, included in the study, completing follow-up, and analysed | | 8 | Line 180: Across all South Dakota secondary school athletic directors (n=177), 112 started (63.3%) and 63 completed (35.6%) the survey. | |
|  |  |  | (b) Give reasons for non-participation at each stage | |  |  | |
|  |  |  | (c) Consider use of a flow diagram | |  |  | |
| Descriptive data | 14* | | (a) Give characteristics of study participants (eg demographic, clinical, social) and information on exposures and potential confounders | | 8-9 | Line 181: The athletic directors were 44.09 + 12.39 years…Most respondents (73.03%, n = 65/89) …had access to AT services… 75% (n = 48/63) indicated the services were part-time…Over 70% (n = 48/68) of respondents indicated the average distance in miles between their school's practice and game venues and the nearest hospital was 15 miles or less. Just over three-fourths (77.78%, n = 49/63) of school districts were in high MHI counties (Table 2.) | |
|  |  |  | (b) Indicate number of participants with missing data for each variable of interest | |  |  | |
|  |  |  | (c) *Cohort study*—Summarise follow-up time (eg, average and total amount) | |  |  | |
| Outcome data | 15* | | *Cohort study*—Report numbers of outcome events or summary measures over time | |  |  | |
|  |  |  | *Case-control study—*Report numbers in each exposure category, or summary measures of exposure | |  |  | |
|  |  |  | *Cross-sectional study—*Report numbers of outcome events or summary measures | | 8 | Line 189: …(73.03%, n = 65/89) reported their school had access to AT services…70% (n = 48/68) of respondents indicated the average distance in miles between their school's practice and game venues and the nearest hospital was 15 miles or less…(77.78%, n = 49/63) of school districts were in high MHI counties…(77.78%, n = 49/63) of schools were in EMS districts with average response times ranging from 13.42-21.65 minutes. | |
| Main results | 16 | | (*a*) Give unadjusted estimates and, if applicable, confounder-adjusted estimates and their precision (eg, 95% confidence interval). Make clear which confounders were adjusted for and why they were included | | 9-11 | Line 189-253: Most respondents (73.03%, n = 65/89) reported their school had access to AT services…  While nearly 91% (n = 49/54) of respondents indicated having a written plan for every practice and game venue, schools without access to AT services were less likely (p = .027 from logistic model with n = 32 and CI (-10.7, -0.79)….  Schools with access to AT services and greater hospital distance were less likely to possess written heat illness guidelines based on logistical regression (p = .02, n = 36, CI = -0.17 -0.017). | |
|  |  |  | (*b*) Report category boundaries when continuous variables were categorized | |  |  | |
|  |  |  | (*c*) If relevant, consider translating estimates of relative risk into absolute risk for a meaningful time period | |  |  | |

Continued on next page

| Other analyses | 17 | Report other analyses done—eg analyses of subgroups and interactions, and sensitivity analyses |  |  |
| --- | --- | --- | --- | --- |
| Discussion | | | | |
| Key results | 18 | Summarise key results with reference to study objectives | 11-18 | Lines 254-408: …almost three-fourths of South Dakota secondary schools receive AT services. This is higher than previously reported… Athletic training services were associated with MHI. This supports prior research…schools that were located in low MHI counties and who did not have access to AT services were less likely to possess written EAPs... This finding was consistent with previous research on secondary school emergency preparedness and access to AT services…Only 33% of respondents in this study indicated their school requires coaches to be CPR certified and trained in the use of AEDs in addition to meeting the SDHSAA requirements; half of these respondents indicated this applied to head coaches only. Our findings were considerably lower than comparable studies from Iowa and Arizona citing the percentage of secondary school coaches (78.1% and 86% respectively) who were both CPR certified and trained in the use of AEDs. |
| Limitations | 19 | Discuss limitations of the study, taking into account sources of potential bias or imprecision. Discuss both direction and magnitude of any potential bias | 18-19 | Lines 410-426: While our survey response rate exceeded 35%, the results are specific to South Dakota and thus difficult to generalize…. MHI as a socioeconomic indicator for income was based on previous research,7 property tax valuation might be more appropriate to an agricultural focused state like South Dakota... South Dakota is divided into 7 different EMS districts that differ in geographical size and population density. Thus, average EMS response time for a district may not accurately reflect the actual response time… |
| Interpretation | 20 | Give a cautious overall interpretation of results considering objectives, limitations, multiplicity of analyses, results from similar studies, and other relevant evidence | 19-20 | Lines 428-457: Considering the challenges of AT access and EMS response times across South Dakota, administrators and secondary school coaches should… would benefit from identifying an emergency planning coordinator…Healthcare systems, county extension services, or the SDHSAA should consider a more active approach to educating… Schools should commit to annual EAP training days… Future research should include novel approaches to EAP education as well as the impact of volunteer EMS services on secondary school emergency preparedness... schools should strongly consider developing policies that require all coaches to secure and maintain CPR certification and AED training... Future research should include barriers to coach CPR certification and AED training in South Dakota…schools may need to consider stronger on-boarding processes for new athletic directors…expanding the requirement for heat illness prevention training to all coaches regardless of season…. Schools should adopt policies and procedures that guide their approach to EHI. This should include timely access to CWI for all practice and game venues and a commitment to assessing core temperature cases of suspected EHI. Future research efforts should include barriers to best practices in EHI recognition and management… |
| Generalisability | 21 | Discuss the generalisability (external validity) of the study results | 18 | Line 410: While our survey response rate exceeded 35%, the results are specific to South Dakota and thus difficult to generalize. |
| Other information | |  | | |
| Funding | 22 | Give the source of funding and the role of the funders for the present study and, if applicable, for the original study on which the present article is based | 20 | Line 458: The authors have no conflicts of interest. This study was supported by South Dakota State University. |

*Give information separately for cases and controls in case-control studies and, if applicable, for exposed and unexposed groups in cohort and cross-sectional studies.

**Note:** An Explanation and Elaboration article discusses each checklist item and gives methodological background and published examples of transparent reporting. The STROBE checklist is best used in conjunction with this article (freely available on the Web sites of PLoS Medicine at http://www.plosmedicine.org/, Annals of Internal Medicine at http://www.annals.org/, and Epidemiology at http://www.epidem.com/). Information on the STROBE Initiative is available at www.strobe-statement.org.
